# Combining with immunotherapy is an emerging trend for local treatment of colorectal cancer liver metastases: a bibliometric analysis

**DOI:** 10.3389/fonc.2025.1490570

**Published:** 2025-04-01

**Authors:** Bin Shao, Ya-Shi Yin, Yi-Nuo Wei, Peng Dong, Hou-Fa Ning, Guang-Zhi Wang

**Affiliations:** ^1^ School of Medical Imaging, Shandong Second Medical University, Weifang, Shandong, China; ^2^ Department of Medical Imaging Center, Affiliated Hospital of Shandong Second Medical University, Shandong Second Medical University, Weifang, Shandong, China

**Keywords:** colorectal cancer, liver metastasis, local treatment, bibliometric analysis, ablation, transarterial chemoembolization, radiation therapy, immunotherapy

## Abstract

**Background:**

A growing body of evidence has demonstrated the expanding role of local treatment in managing colorectal cancer liver metastases (CRCLM). To identify current research trends and forecast future directions, we conducted a bibliometric analysis to examine global collaboration patterns and academic influence across countries, institutions, journals, and authors.

**Materials and methods:**

Relevant articles and reviews on CRCLM local therapies were systematically retrieved from the Web of Science Core Collection. The bibliometric package in R software and VOSviewer software were used to analyze countries, institutions, journals, authors, and keywords. The research status and key areas of local treatment of colorectal cancer liver metastases were analyzed by keywords.

**Results:**

The analysis encompassed 2,695 articles published between 2008 and 2023. The United States emerged as the leading contributor, with Memorial Sloan Kettering Cancer Center producing the highest number of publications (n=178). Among journals, *Annals of Surgical Oncology* ranked first in publication volume, while *Journal of Vascular and Interventional Radiology* achieved the highest citation count. The local treatment modalities for CRCLM included transarterial therapies (radioembolization and chemoembolization), hepatic artery infusion chemotherapy and immunotherapy, imaging guidance methods, hepatectomy and survival, and ablation and stereotactic body radiotherapy. Recent studies highlighted ablations, microspheres, and immunotherapy as key research areas, with thematic mapping identifying immunotherapy as an emerging niche field.

**Conclusion:**

CRCLM local treatment research focuses on integrating local and systemic therapies. Preclinical studies, RFA with anti - PD - 1 agents, show enhanced anti - tumor immunity and survival. While the synergy of local and immunotherapy is confirmed, large - scale clinical evidence is still needed. Thus, cross - disciplinary cooperation is urgently required to boost translational medical research.

## 1 Introduction

Colorectal cancer (CRC) stands as the second leading cause of cancer mortality worldwide, and it ranks as the third most prevalent oncological on a global scale (1). Metastasis occurs in 20-50% of patients with CRC. The most common site of metastasis is the liver, followed by the lungs, peritoneum, and distant lymph nodes (2). Approximately 14.5-56.4% of CRC patients experience synchronous metastasis, and 10.3–19.6% of patients develop metachronous metastasis from the primary colorectal cancer (2–6). Hepatic resection serves as the standard therapeutic approach for colorectal cancer liver metastases, however, only 6.1% to 25.4% of patients ultimately undergo liver metastasis resection (2, 3, 6). In 2016, ESMO guidelines put forward the concept of oligometastatic disease (OMD), which is in the middle of the state between the limitation of the primary tumor and extensive metastases, 2-3 transfer parts or metastases number 5 or less. These OMD patients can achieve a good prognosis by achieving No Evidence of Disease (NED), and emphasize the significance of local treatment in these patients (7). At present, local treatment strategies focus on three major directions: ablation technology, such as radiofrequency ablation (RFA) or microwave ablation (MWA), can achieve precise tumor inactivation and provide effective local control for unresectable lesions. Stereotactic body radiation therapy (SBRT) provides precise radiotherapy for patients with unresectable lesions (8–10), while local-regional treatment represented by hepatic arterial infusion chemotherapy (HAIC) improves the efficiency of drug distribution through targeted drug delivery strategy (11, 12). As more evidence emerges, these local treatments are being recognized for their ability to achieve local control and contribute to prolonged liver progression-free survival (PFS) (13–15). In recent years, a growing number of studies have shown that local treatment of CRCLM plays an important role in tumor treatment initiated by multidisciplinary teams. Notably, local therapies can modulate the immune system through distinct mechanisms, thereby eliciting systemic immune responses (16, 17). For instance, RFA enhances the exposure of tumor-associated antigens, which improves dendritic cell presentation efficiency. When combined with PD-1/PD-L1 inhibitors, this approach significantly augments distal antitumor effects (18). However, the antitumor immune response triggered by standalone local therapy remains limited in intensity, failing to achieve systemic tumor eradication (16). This mechanistic limitation provides a theoretical rationale for combination therapy. Synergizing with systemic treatments such as immune checkpoint inhibitors creates a collaborative mode wherein local ablation activates an *in situ* vaccine effect, while systemic immunotherapy enhances distal tumor killing. Bibliometrics, an interdisciplinary field of science, applies mathematical and statistical methods to analyze literature in specific research areas, with the aim to revealing the advancement of knowledge and research trends (19). Shi et al. (20) discussed the surgical treatment, chemotherapy and auxiliary diagnosis of CRCLM, and Jin et al. (21) analyzed the management evolution of CRCLM. To our knowledge, this is the first bibliometric analysis to systematically map the integration of local and immunotherapies in CRCLM management. Unlike past reviews focused on surgery or systemic therapy alone, our study uniquely reveals new trends, research hotspots, and translational bottlenecks in local treatment strategies to promote CRCLM paradigm innovation.

## 2 Materials and methods

### 2.1 Data acquisition source and search strategies

Related articles were retrieved from the Web of Science Core Collection (WoSCC) database. The retrieval was performed until November 22, 2023, and the exported records include full-text records and references in plain text format. Details of the specific extracted data are in **Supplementary Table S1**. The data used in this study were obtained from the WoS database; hence, ethical approval does not apply to this study.

### 2.2 Data screening and preprocessing

**Figure 1:**
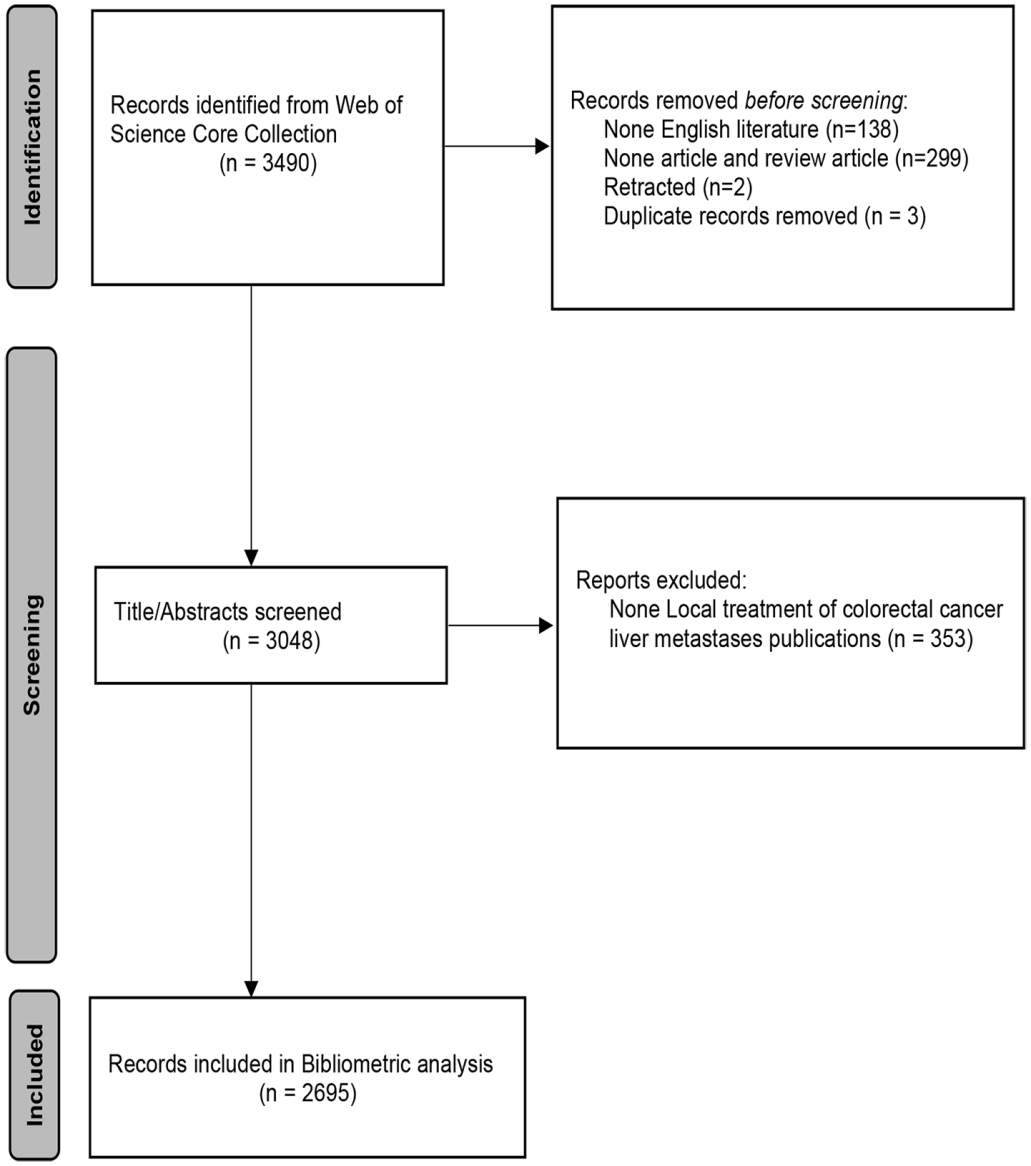
Flow diagram of the literature selection process.

Document types were limited to “article,” “review,” and “early access.” The publication period was limited to the period January 2008 to November 2023. Only publications in English were included. Exclusion criteria were irrelevant publications; “meeting abstracts,” “proceeding paper,” “editorial material,” “letter,” “book chapters,” and “correction”; duplicated or retracted publications. Two investigators (SB and WYN) independently reviewed the bibliometric indicators of publications. If there were disagreements, they were discussed and resolved with the assistance of a third investigator (WGZ). The flow chart was shown in **Figure 1**.

### 2.3 Bibliometric analysis

Two types of bibliometric software R bibliometrix (version 4.1.3) and VOSviewer (version 1.6.19) were used to conduct analysis, and Microsoft Office Excel 2021 software (Microsoft, Redmond, WA, USA) were used to produce annual production and citation trend graphs. Our research team comprises certified specialists in advanced bibliometric analysis, experts proficient in specialized software tools, and medical doctors with extensive clinical experience in oncology and interventional radiology. Bibliometrix is a R-tool, especially designed in the R language, for comprehensive bibliometric analysis (22). The h-index, g-index and m-index were used to measure the academic influence of the authors. The h-index takes into account both how many papers they have published and how often those papers have been cited (23). R bibliometrix was used to analyze the research output, average annual citation frequency, country, institution, journal, author, author keywords and thematic map in the field of local treatment of CRCLM.

VOSviewer was used to construct the cooperation network of countries, institutions and authors, the coupling network of journals, the keyword clustering map and the overlay map, which is a visualization construction and visualization tool widely used in bibliometric research (24).

## 3 Result

### 3.1 The trend in publications and citations

**Figure 2:**
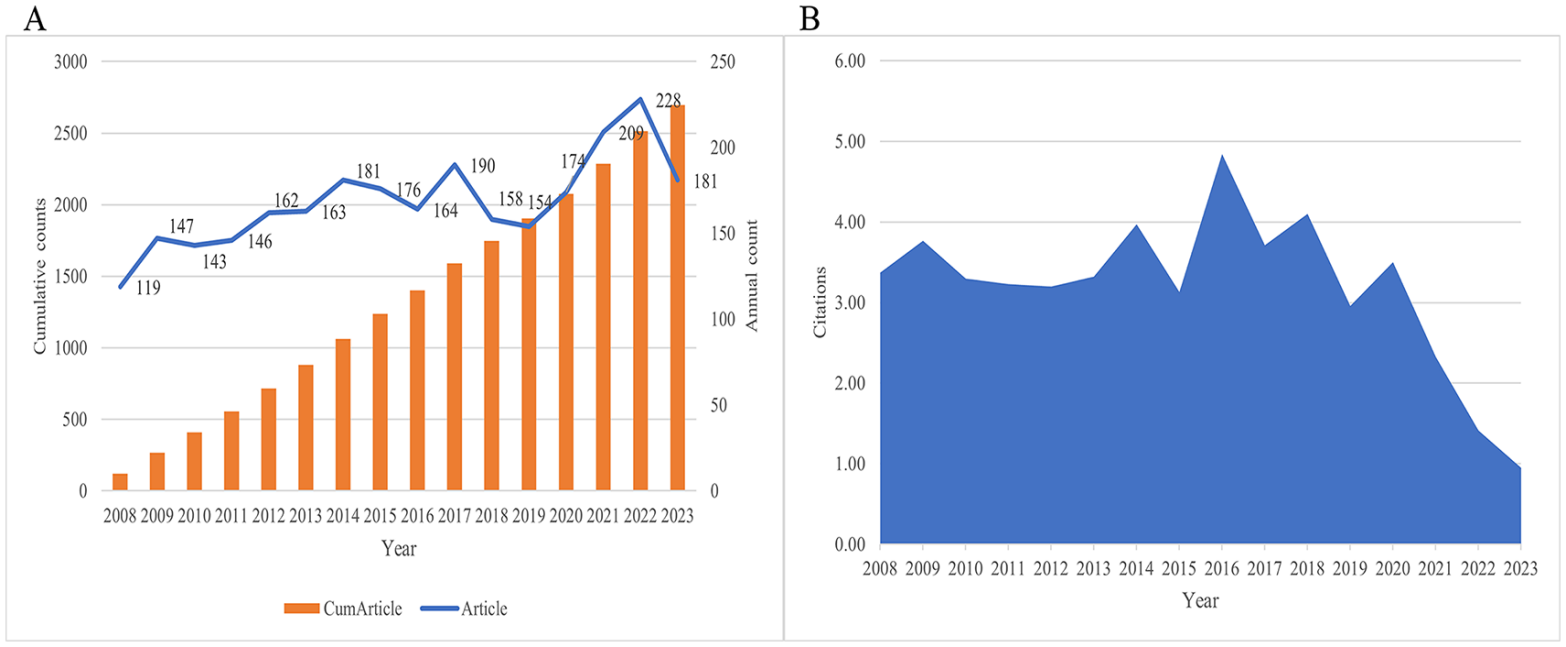
Trends in the number of publications and citations. **(A)** A line chart of the number of publications published each year, 2008-2023. **(B)** A line chart showing the average number of citations per year.

A total of 2695 articles on local treatment of CRCLM were retrieved from 2008 to 2023, including 2156 articles and 539 reviews through December 22, 2023. Since 2008, the number of annual publications on local treatment of CRCLM showed an overall upward trend (**Figure 2A**). 228 studies were published in 2022, making it the year with the most publications in recent years. **Figure 2B** shows that the average citation count was highest in 2016 at 5.44. Due to the study’s deadline window, it does not truly reflect academic output in 2023. At the time of our search, there were a total of 181 publications with an average citation count of 1.90 (**Supplementary Table S2**).

### 3.2 Countries

**Figure 3:**
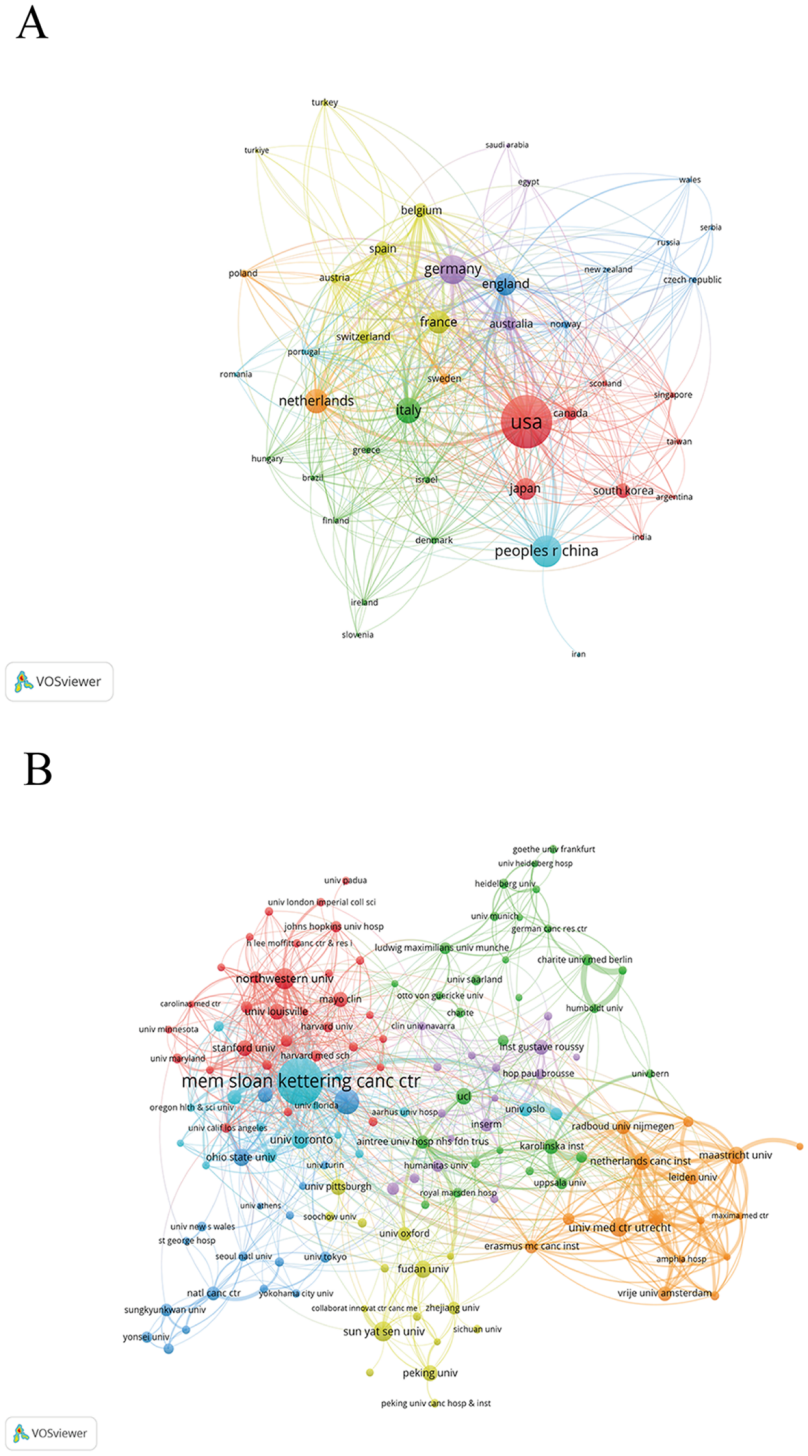
Analysis of collaboration networks between countries/regions and institutions. **(A)** Node size indicates the number of published papers, color indicates the cluster to which the country belongs, and line thickness indicates the degree of cooperation between countries. **(B)** Node size indicates the number of papers published by the institution, color indicates the cluster to which the institution belongs, and line thickness indicates the degree of cooperation.

Researchers from 52 countries have collectively published 2,695 articles on local treatments for CRCLM. The five countries with the highest number of published articles were the United States (696), China (340), Germany (232), Italy (182) and the Netherlands (171). The top 10 high yield countries published 2,208 articles, accounting for 82% of the number of published articles (**Table 1**). Additionally, the United States recorded the highest total number of citations at 26,521 times. This leadership position of the United States may be attributed to extensive funding and multidisciplinary collaborations, which have driven advancements in the field. As Asian countries, Japan (27.40), China (11.90), and South Korea (19.80) have much lower average citations compared to other countries (**Supplementary Table S3**). A collaborative network of 43 countries for local treatment of CRCLM was established via VOSviewer and seven clusters were generated (**Figure 3A**). The United States holds a prominent position within the collaborative network and has established close collaboration with 38 other nations worldwide. There are also important collaborations between Germany, the United Kingdom, France and other countries.

**Table 1:** Top 10 high-yield countries.

Rank	Country	Articles	TC	Average Article Citations
1	USA	696	26521	38.1
2	CHINA	340	4040	11.9
3	GERMANY	232	8393	36.2
4	ITALY	182	5339	29.3
5	NETHERLANDS	171	5742	33.6
6	JAPAN	159	4350	27.4
7	FRANCE	144	6699	46.5
8	UNITED KINGDOM	135	4594	34
9	KOREA	88	1745	19.8
10	AUSTRALIA	61	2031	33.3

### 3.3 Institution analysis

The research area includes a total of 3,213 research institutions. The top three in terms of the number of articles published are Memorial Sloan Kettering Cancer Center (MSKCC) (178 articles), University of Texas MD Anderson Cancer Center (64 articles) and Northwestern University (48 articles) from the United States (**Table 2**). They are also in the top three with 7,942, 2,677 and 2,562 citations respectively. The collaborative network of 143 institutions in this area of local treatment of CRCLM demonstrated the degree and strength of institutional collaboration (**Figure 3B**). MSKCC works closely with respected academic institutions worldwide and promotes extensive collaboration. MSKCC has the largest number of links, with a total of 58, followed by Northwestern University (37) and Stanford University (37), which have the most links with other institutions (**Supplementary Table S4**). MSKCC (165), Netherlands Cancer Institute (95), and University of Amsterdam (91) received the highest total link strength (**Supplementary Table S4**).

**Table 2:** Top 10 institutions with the most articles.

Rank	Institutions	Number of articles	Total Citations	Average citations	Country
1	Memorial Sloan Kettering Cancer Center	178	7942	44.618	USA
2	University of Texas MD Anderson Cancer Center	64	2677	41.8281	USA
3	Northwestern University	48	2562	53.375	USA
4	Sun Yat-sen University	42	576	13.7143	China
5	University Medical Center Utrecht	40	1115	27.875	Netherlands
6	University of Toronto	38	1488	39.1579	Canada
7	Maastricht University	34	708	20.8235	Netherlands
8	Netherlands Cancer Institute	34	480	14.1176	Netherlands
9	Fudan University	33	525	15.9091	China
10	Ohio State University	33	1414	42.8485	USA

### 3.4 Main source journals and co-citation analysis

The five journals with the highest publication frequency are *Annals of Surgical Oncology* (100), *Cardiovascular and Interventional Radiology* (74), *Journal of Surgical Oncology* (63), *EJSO* (60), and *Cancers* (57) (**Supplementary Table S5**). Among the top 10 journals with the highest number of publications, four journals had an average citation frequency of more than 30 times. The *Journal of Vascular and Interventional Radiology* had the highest average citation frequency (47.06), followed by *European Radiology* (36.02), *Annals of Surgical Oncology* (33.96), and *World Journal of Gastroenterology* (33.96). The total citations of these journals were 2,447, 1,549, 3,396 and 1,630 respectively.

A total of 2695 publications on local treatment of CRCLM were published in 491 journals. The literature coupling network of journals on local treatment of CRCLM was constructed by VOSviewer (**Supplementary Figure S1**). The network is based on bibliographic coupling and includes 65 journals, with most publications being *Annals of Surgical Oncology* (100 articles), followed by *Cardiovascular and Interventional Radiology* (74 articles) and *Journal of Surgical Oncology* (63 articles).

### 3.5 Author analysis

We identified the top 20 most influential authors based on their h-index (**Supplementary Table S6**). Among all authors, Kemeny NE has the highest h-index (30), followed by D’angelica MI (29) and Jarnagin WR (29). These top three authors are all affiliated with MSKCC. Kemeny NE is a leader in h-index, g-index, and publication output. Authors’ collaborative network analysis was created using VOSviewer (**Supplementary Figure S2**). These authors were divided into 17 clusters, among which we identified several prominent research teams, including Kemeny NE, Kingham TP, Sofocleous CT, and Salem R. In the upper right corner of **Supplementary Figure S2**, a relatively independent research team led by Meijerink, M.R, has published a large number of articles on ablation of CRCLM in recent years.

### 3.6 Analysis of keywords and research trends

**Figure 4:**
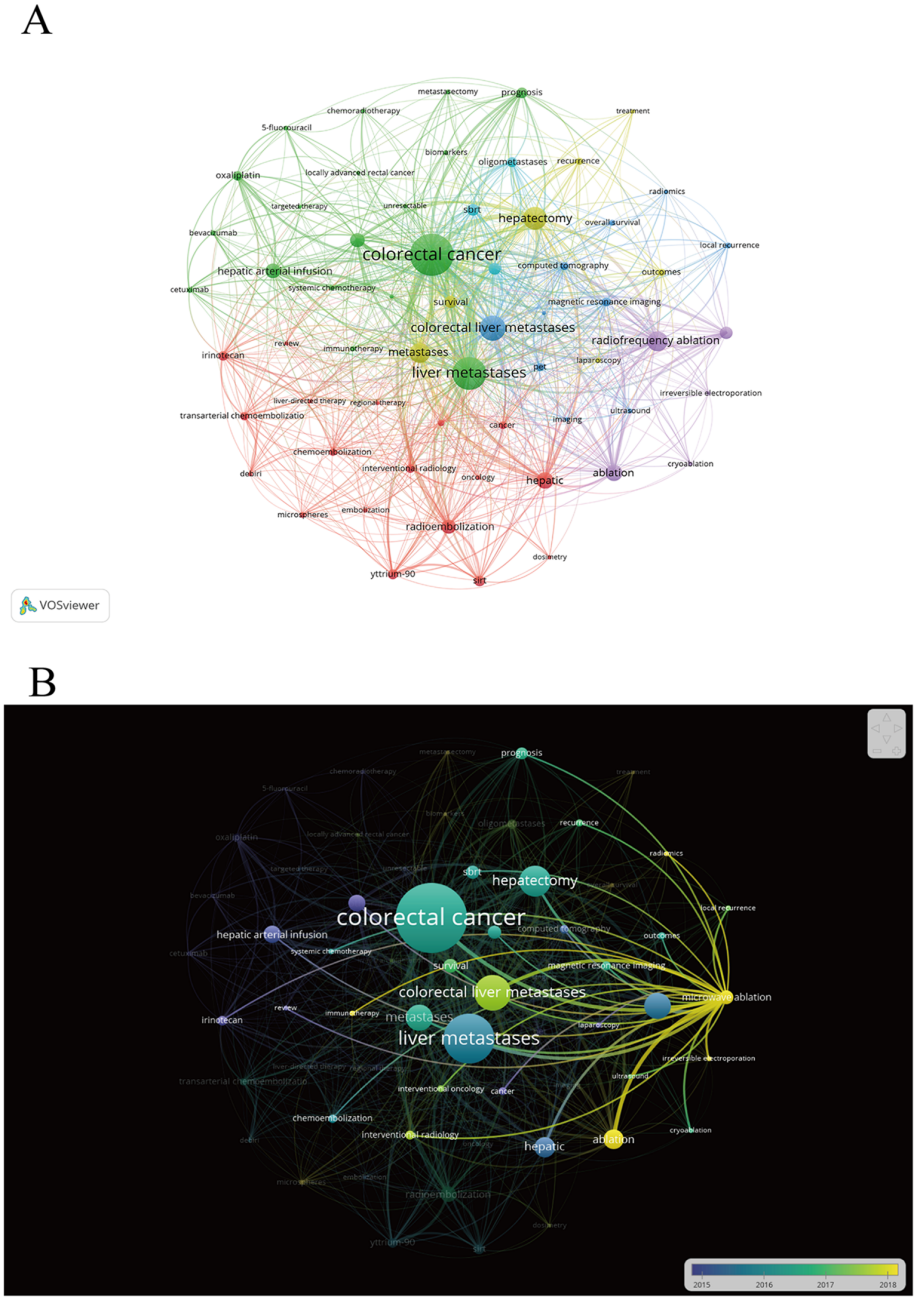
Keyword visualization. **(A)** Cluster analysis of keywords. The node size indicates the frequency of occurrence of the keyword; the line between nodes indicates the existence of a co-occurrence relationship; the keyword co-occurrence network is divided into 3 color - coded clusters according to the evolution of research hotspots. **(B)** Visualization of keywords’ overlay. The color of a keyword represents the average publication time of articles containing it.

Potential research hotspots were identified by analyzing the co-occurrence of keywords. A total of 3249 author keywords were extracted using VOSviewer software, and after synonym conversion and singular/plural substitutions, 61 keywords appeared more than 12 times. The most frequently used keyword is colorectal cancer, liver metastases, hepatectomy, colorectal liver metastases, and radiofrequency ablation (**Supplementary Table S7**). Each color represents a cluster, all keywords can be divided into 6 clusters (**Figure 4A**). Keywords that are highly correlated form clusters of the same color. Cluster 1 (red) consists of 18 co-occurring words related to radioembolization and transarterial chemoembolization. Cluster 2 (green) consists of 18 terms related to HAIC and immunotherapy for CRCLM. Cluster 3 (blue) focuses on the use of imaging guidance methods. Cluster 4 (yellow) consists of 7 keywords related to hepatectomy and survival of CRCLM. Cluster 5 (purple) consists of 5 keywords related to ablation techniques. The final cluster (cyan) contained three keywords related to SBRT. Based on the analysis of co-occurrence clustering of keywords, further analysis of keyword overlay visualization is carried out (**Figure 4B**). The keywords that appeared frequently after 2018 include ablation, microwave ablation, irreversible electroporation, microspheres, immunotherapy, etc. These keywords that appeared frequently in recent years may become future research focuses.

A thematic map created by R bibliometrix showed that authors’ keywords were grouped according to their relevance and development in the research area (**Supplementary Figure S3**). The motor themes in the upper right corner appear as “hepatic arterial infusion” and “transarterial chemoembolization.” These are developed and mature studies. The basic themes in the lower right corner, including “ablation” and “radioembolization,” are the basic themes of this study and can be further explored and developed. In addition, there are three groups of niche themes in the upper left, including “Immunotherapy,” suggesting that the advanced internal connections are helpful for this research area, local treatment in combination with systemic therapy, especially in combination with immunotherapy, provides an effective strategy for the treatment of CRCLM. Bottom left Emerging or Declining themes include “magnetic resonance imaging” and “sbrt”, which are considered emerging topics and are gradually having an impact on the research field.

## 4 Discussion

In the past 16 years, the number of articles on local treatment of CRCLM has shown a rapid growth trend, reaching a peak in 2022, indicating the increasing importance of local treatment. The peak of the average number of citations per year was in 2016, which can be attributed to the ESMO consensus guidelines for the management of patients with metastatic colorectal cancer. These guidelines were published in the *Annals of Oncology* by Van Cutsem, E, and they provide a body of evidence-based recommendations to support the treatment and care of patients (7). The United States demonstrates prominent academic leadership in the management of CRCLM, attributable to sustained research investment, extensive global collaboration networks, and advanced technology translation capabilities. As a central research hub, MSKCC has established collaborations with 58 leading institutions worldwide. Journal analysis reveals that *Annals of Surgical Oncology* and *Journal of Vascular and Interventional Radiology* hold authoritative status in interventional therapeutics, with their publications focusing on ablation technique optimization and complication mitigation in Yttrium-90 radioembolization, providing key academic reference for scholars. Notably, Jarnagin WR and D’Angelica MI and Kemeny NE of MSKCC have made outstanding contributions to HAIC research in CRCLM and are preferred candidates for international collaboration and frontier exchange.

The bibliometric analysis categorized research domains into six thematic clusters through keyword co-occurrence mapping: Transarterial embolization (red cluster), HAIC and immunotherapy (green), Image-guided interventions (blue), Hepatectomy and survival outcomes (yellow), Ablation techniques (purple), SBRT (cyan). The frequent recent appearance of immunotherapy - related keywords, and its classification as a niche theme in the thematic map, indicate it has formed a specialized knowledge cluster but isn’t yet fully integrated into the core treatment network. This may reflect rapid advancements in specific immunotherapy paths like immune checkpoint inhibitors and CAR - T cell therapy, though clinical application limitations remain. Despite its current low centrality, with growing immunotherapy combination trials, local treatment combined with immunotherapy may evolve into a core research topic.

RFA is currently the most well-studied evidence in patients with unresectable CRCLM and is used more frequently than other ablative modalities. According to the keyword overlay, MWA has been used more and more frequently in recent years. Other ablation methods, like Cryoablation (CA), Irreversible Electroporation Therapy (IRE), and High Intensity Focused Ultrasound (HIFU), also benefit CRCLM patients. Numerous clinical trials have confirmed that local ablation is a safe and effective treatment for CRCLM. In a multicenter phase 3 clinical trial, thermal ablation was comparable to surgical resection for local control, and conversion of surgical resection to thermal ablation reduces complications and improves local control without affecting OS and PFS (25). In addition, RFA combined with systemic therapy has emerged as a potential treatment modality. In the CLOCC trial, RFA combined with systemic therapy had a significantly higher 3-year PFS rate (27.6%) than systemic therapy alone (10.6%) (P = 0.025). The median overall survival (OS) was higher in the combination therapy group than in the systemic therapy alone (P = 0.22) (26). Retrospective studies have shown that MWA or combined with hepatectomy for liver metastases results in good long-term survival, while for small tumors (<3 cm) and tumors distant from blood vessels (27). 293 patients underwent cryoablation or cryoablation combined with surgery. The results showed that survival rates at 1, 3, 5, and 10 years were 87%, 41.8%, 24.2%, and 13.3%, respectively. The DFS rates for the same periods were 37.9%, 17.2%, 13.4%, and 10.8%, respectively. Intrahepatic recurrence was observed in 161 patients (28).

Ablation therapy induces localized coagulative necrosis while simultaneously augmenting anti-tumor immunity through multiple mechanisms: (1) enhanced tumor antigen exposure and immunogenicity, (2) activation of antigen-presenting cells, (3) expansion of tumor-specific T-cell populations, and (4) alleviation of immunosuppressive microenvironment. However, ablation monotherapy fails to achieve complete tumor eradication due to residual micrometastases and adaptive immune resistance. By enhancing the response of RFA to local immune initiation, it may be effective in assisting the generation of distant effects, i.e. local treatment-induced control of tumors in other parts of the body, and for larger lesions, the combination of RFA and local immunomodulation may have a synergistic effect (29). In mouse models, combining RFA with anti-PD-1 antibody treatment significantly boosts T - cell immunity, improves antitumor effects, and extends survival. These findings provide compelling preclinical rationale for clinical trials investigating RFA-PD-1/PD-L1 blockade combinations in metastatic CRC (30). The expression of LAG3 has been found to be upregulated after MWA, and an experiment in a mouse model suggests that introducing LAG3 blockade into MWA delays tumor progression and prolongs survival. MWA in combination with immune checkpoint inhibitor therapy is a potentially effective regimen for the treatment of CRCLM (31). In terms of immunotherapy, the tumor can serve as its own antigen vaccine after ablation, with cryoablation achieving a stronger immune response compared to other thermal ablation methods such as RFA (32, 33). Other ablation techniques, such as HIFU and IRE, induce an overall weak immune response, and the clinically effective tumor-specific immune response is relatively weak (34–36).

The recent increasing use of SBRT in patients with CRCLM who are unsuitable or difficult to undergo liver resection or RFA, SBRT emerging as an effective and safe extracorporeal radiotherapy technique with good local control rates (37, 38). Petrelli et al. conducted a systematic review of a total of 656 patients with CRCLM approximately 3 cm in size. Their results indicated that SBRT provided long-term local control and 2-year PFS rates of 59% and 56%, respectively (39). Growing evidence supports the emerging roles of OMD-directed therapies, with both SBRT and SIRT demonstrating promising potential as locoregional treatment options for patients with unresectable CRCLM (40). SBRT can induce systemic antitumor responses, including regression of untreated distant metastases, a phenomenon termed the abscopal effect. However, SBRT also triggers immunosuppressive mechanisms, such as increased TGF-β secretion and upregulated PD-L1 expression, which may counteract its immunostimulatory potential (41). To address this dual role, combining SBRT with immune checkpoint inhibitors offers a strategy to synergistically enhance antitumor efficacy. Current clinical trials are exploring synergistic strategies to optimize SBRT-immune therapy combinations. Two clinical trials have optimized the SBRT - immunotherapy combination. One Ib/II - phase trial (NCT03436563) studied m7824, a TGF-β/PD-L1 dual inhibitor, with radiotherapy for high MSI mCRC, aiming to block immunosuppressive pathways and enhance radiation - triggered immune activation. Another Ib - phase trial (NCT02837263) assessed SBRT plus pembrolizumab for CRLM, focusing on using SBRT’s immunogenic priming effect to overcome PD - L1 - mediated resistance. Though the m7824 trial didn’t meet expectations and the Phase Ib Trial results are unpublished, ongoing research is refining patient selection and treatment plans. Future studies are crucial to validate these combination strategies’ potential.

In addition, locoregional treatments such as intra-arterial therapies, TACE is a minimally invasive procedure that blocks the blood vessels of a tumor with liver-directed chemotherapy, causing maximum exposure of the agent to the ischemic environment (11). Two randomized trials investigated the use of TACE for CRCLM using irinotecan-based drug-eluting microspheres (DEBIRI), but both studies had significant limitations in their design and analysis (42). HAIC is a commonly used treatment modality in which intraarterial ports or pumps are surgically or percutaneously placed to enhance the therapeutic effect of hepatic metastatic colorectal cancer by delivering high concentrations of drugs to the liver (43). In a multicenter randomized controlled trial, researchers found that patients’ median OS improved significantly when HAIC was combined with systemic chemotherapy (20 months vs 14 months, P = 0.003), indicating that HAIC combined with systemic therapy holds great therapeutic promise (11, 44). TACE exerts dual immunomodulatory effects in tumor therapy. While embolization-induced tumor necrosis reduces tumor volume and alleviates immune suppression by decreasing immunosuppressive factors (45), it simultaneously triggers systemic immune activation through phenotypic changes in surrounding immune cells (46). This duality highlights the therapeutic potential of combining TACE with immunotherapy. Clinical studies demonstrate promising outcomes: A single-center trial showed regorafenib plus DEB-TACE significantly improved outcomes in colorectal cancer patients with liver metastases versus regorafenib alone (median PFS 7.6 vs 4.1 months, OS 15.7 vs 9.2 months; ORR 35.3% vs 7.1%) (47). The 2021 ASCO-reported HAIC-regorafenib combination achieved 22.2-month OS and 51.3% ORR in mCRC with hepatic metastases (48). These findings position locoregional therapies as valuable components in combination strategies for pMMR/MSS mCRC with hepatic metastases, warranting further investigation into immunotherapy-TACE/HAIC integrations.

TARE/SIRT is a specialized radiation technique that involves the precise delivery of radioactive microparticles directly into the branches of the hepatic artery that supply the liver (49). Failed chemotherapeutic options for liver-limited metastases patients, TARE with Y-90 resin microspheres has proven to be a promising treatment approach. In a small randomized study, TARE with Y-90 resin microspheres was shown to prolong tumor progression and liver progression (50). Two early-phase studies explored combining SIRT with immunotherapy. A single-arm phase II trial (N=Microsatellite Stable mCRC patients) testing SIRT plus durvalumab was terminated early due to treatment futility, with all participants showing hepatic disease progression (51). A separate phase Ib trial investigating intra-arterial anti-CEA CAR T-cell therapy combined with SIRT in treatment-refractory CEA+ liver metastases reported a median survival of 8 months (52). Current evidence does not support clinical benefits from SIRT-immunotherapy combinations, though further investigation remains warranted to fully evaluate potential therapeutic synergies (53).

Image-guided methods such as MRI, CT, PET and ultrasound have made navigation increasingly precise and ablation technology has become more widespread. The follow-up methods for local recurrences and prognosis are becoming more and more numerous. With the development of radiomics, the treatment methods for liver metastases in colorectal cancer are becoming more and more refined. While conventional CT of the abdomen is used for the diagnosis and staging of liver metastases, MRI has emerged as the preferred method for accurately determining their number and location. Percutaneous, laparoscopic, or intraoperative methods under CT or MRI guidance can be used to locate and monitor liver metastases while assessing treatment effectiveness (54, 55). PET scans are particularly valuable in patients with elevated tumor markers but no evidence of metastatic disease or when evaluating the extent of potentially resectable metastases (2, 37, 56).

Despite the promising synergy between local and immunotherapeutic approaches in managing CRCLM, the lack of standardized protocols in current clinical practice significantly hinders their widespread adoption and efficacy optimization. Key challenges include the absence of consensus on optimal dosing and timing of immunotherapy and interventional therapies, necessitating personalized adjustments. While most TACE-immunotherapy trials administer treatments concurrently or sequentially, emerging evidence suggests delaying interventional therapy until progression might mitigate hepatic toxicity risks. Current immune phenotyping (“cold/hot tumors”) remains limited by pathological variability, requiring integration of radiomics and liquid biomarkers for precise prediction (57). To address these gaps, future efforts should prioritize: Evidence-based standardization of treatment sequences, dosing, and safety monitoring; Implementation of imaging-guided technologies for real-time immune response assessment; International multicenter phase III trials to validate survival benefits and cost-effectiveness. Only through harmonizing standardization with personalized strategies can maximal clinical benefits be achieved for CRCLM patients undergoing combined therapies.

However, this study also has some limitations. First, the study was confined to English-language literature indexed in the Web of Science database. This linguistic limitation, particularly the absence of non-English publications (e.g., Japanese and Korean scholarly works), may compromise the generalizability of the findings. Second, some newly published high-quality papers may not be evaluable due to low citation frequency. Finally, most of the studies on local treatment combined with immunotherapy are animal experiments, and there is a lack of clinical trial cases. Despite these limitations, bibliometric analysis may provide a better understanding of research trends and hotspots in local treatment of CRCLM.

## 5 Conclusion

The current research trends in localized treatment for CRCLM emphasize the integration of localized and systemic therapies. Preclinical experiments, such as those employing RFA combined with anti-PD-1 agents, have shown a marked enhancement of anti-tumor immunity and extended survival in preclinical models. Preclinical studies have confirmed the synergistic potential of local and immunotherapy, but large - scale clinical evidence is still needed. There is an urgent need for cross - disciplinary cooperation to boost translational medical research.

## Data Availability

Publicly available datasets were analyzed in this study. This data can be found here: Web of Science Core Collection.
