# Supplementary material for: Combining with immunotherapy is an emerging trend for local treatment of colorectal cancer liver metastases: a bibliometric analysis
Source: Front Oncol. 2025 Apr 1;15:1490570. doi: 10.3389/fonc.2025.1490570 (PMC11996661; doi:10.3389/fonc.2025.1490570)
Supplement: Supplementary file 1 [file Table1.docx]

Supplementary Material

# Supplementary Tables

**Table S1.** Detailed search strategy

| #1 | TS=(“Colorectal cancer” OR “colorectal neoplasm” OR “Colon cancer” OR “rectal cancer”) |
| --- | --- |
| #2 | TS=(“liver metastasis” OR “hepatic metastasis”) |
| #3 | #1 AND #2 |
| #4 | TS=(“locoregional therapy” OR “local therapy” OR “local treatment” OR “local ablation” OR “ablation” OR “ablation technique” OR “Cryoablation” OR “cryosurgery” OR “cryotherapy” OR “radiofrequency ablation” OR “RFA” OR “microwave ablation” OR “MWA” OR “Irreversible Electroporation Therapy” OR ”IRE” OR “High Intensity Focused Ultrasound” OR “HIFU” OR “Laser Ablation” OR “LA” OR “chemical ablation” OR “Ethanol” OR “acetic acid” OR “hepatic arterial infusion chemotherapy” OR “hepatic artery infusion chemotherapy” OR “HAIC” OR “hepatic arterial infusion” OR “hepatic artery infusion” OR “HAI” OR “transarterial infusion” OR “Transcatheter arterial infusion” OR “TAI” OR “transarterial chemoembolization” OR “TACE” OR “transcatheter arterial embolization” OR “transarterial embolism” OR “TAE” OR “radiotherapy” OR “radiation therapy” OR “radiation treatment” OR “stereotactic body radiation therapy” OR “SBRT” OR “Radioembolization” OR “transarterial radioembolization” OR “TARE” OR “Yttrium 90” OR “Y-90” OR “90Y” OR “selective internal radiation therapy” OR “SIRT” OR “brachytherapy”). |
| #5 | #3 AND #4 |

**Table S2.** The trend in publications and citations

| **Year** | **N** | **Mean TC per Year** |
| --- | --- | --- |
| 2008 | 119.00 | 3.58 |
| 2009 | 147.00 | 4.02 |
| 2010 | 143.00 | 3.54 |
| 2011 | 146.00 | 3.48 |
| 2012 | 162.00 | 3.47 |
| 2013 | 163.00 | 3.63 |
| 2014 | 181.00 | 4.36 |
| 2015 | 176.00 | 3.48 |
| 2016 | 164.00 | 5.44 |
| 2017 | 190.00 | 4.24 |
| 2018 | 158.00 | 4.78 |
| 2019 | 154.00 | 3.55 |
| 2020 | 174.00 | 4.37 |
| 2021 | 209.00 | 3.11 |
| 2022 | 228.00 | 2.12 |
| 2023 | 181.00 | 1.90 |
|  |  |  |

N: Number of articles per year

Mean TC per Year: Yearly average number of times each document has been cited.

**Table S3.** The number of publications and the citations of all countries

| **Country** | **Articles** | **TC** | **Average Article Citations** |
| --- | --- | --- | --- |
| USA | 696 | 26521 | 38.10 |
| CHINA | 340 | 4040 | 11.90 |
| GERMANY | 232 | 8393 | 36.20 |
| ITALY | 182 | 5339 | 29.30 |
| NETHERLANDS | 171 | 5742 | 33.60 |
| JAPAN | 159 | 4350 | 27.40 |
| FRANCE | 144 | 6699 | 46.50 |
| UNITED KINGDOM | 135 | 4594 | 34.00 |
| KOREA | 88 | 1745 | 19.80 |
| AUSTRALIA | 61 | 2031 | 33.30 |
| CANADA | 52 | 1303 | 25.10 |
| SPAIN | 50 | 1190 | 23.80 |
| SWITZERLAND | 39 | 3356 | 86.10 |
| SWEDEN | 39 | 690 | 17.70 |
| BELGIUM | 36 | 1196 | 33.20 |
| NORWAY | 28 | 732 | 26.10 |
| TURKEY | 25 | 404 | 16.20 |
| POLAND | 25 | 184 | 7.40 |
| AUSTRIA | 24 | 602 | 25.10 |
| DENMARK | 17 | 477 | 28.10 |
| GREECE | 17 | 300 | 17.60 |
| CZECH REPUBLIC | 15 | 75 | 5.00 |
| ROMANIA | 11 | 83 | 7.50 |
| ISRAEL | 10 | 308 | 30.80 |
| SINGAPORE | 10 | 246 | 24.60 |
| INDIA | 10 | 78 | 7.80 |
| BRAZIL | 9 | 66 | 7.30 |
| IRELAND | 7 | 162 | 23.10 |
| FINLAND | 7 | 62 | 8.90 |
| SLOVENIA | 6 | 525 | 87.50 |
| HUNGARY | 6 | 13 | 2.20 |
| IRAN | 5 | 77 | 15.40 |
| ARGENTINA | 4 | 57 | 14.20 |
| PORTUGAL | 3 | 51 | 17.00 |
| SERBIA | 3 | 13 | 4.30 |
| NEW ZEALAND | 2 | 127 | 63.50 |
| LITHUANIA | 2 | 15 | 7.50 |
| SLOVAKIA | 2 | 8 | 4.00 |
| CROATIA | 2 | 8 | 4.00 |
| SAUDI ARABIA | 2 | 6 | 3.00 |
| CYPRUS | 1 | 133 | 133.00 |
| MOROCCO | 1 | 60 | 60.00 |
| BULGARIA | 1 | 38 | 38.00 |
| MALAYSIA | 1 | 21 | 21.00 |
| PANAMA | 1 | 10 | 10.00 |
| EGYPT | 1 | 9 | 9.00 |
| MEXICO | 1 | 7 | 7.00 |
| RUSSIA | 1 | 3 | 3.00 |
| PAKISTAN | 1 | 3 | 3.00 |
| GEORGIA | 1 | 3 | 3.00 |
| LEBANON | 1 | 2 | 2.00 |
| JAMAICA | 1 | 1 | 1.00 |

**Table S4.** The detailed information of the institutional partnership

| **Institutions** | **Cluster** | **Links** | **Total link strength** | **Number of articles** | **Total Citations** | **Average citations** |
| --- | --- | --- | --- | --- | --- | --- |
| Mem sloan kettering canc ctr | 6 | 58 | 165 | 178 | 7942 | 44.618 |
| Univ texas md anderson canc ctr | 3 | 33 | 58 | 64 | 2677 | 41.8281 |
| Northwestern univ | 1 | 37 | 87 | 48 | 2562 | 53.375 |
| Sun yat sen univ | 4 | 17 | 29 | 42 | 576 | 13.7143 |
| Univ med ctr utrecht | 7 | 22 | 67 | 40 | 1115 | 27.875 |
| Univ toronto | 6 | 29 | 54 | 38 | 1488 | 39.1579 |
| Netherlands canc inst | 7 | 25 | 95 | 34 | 480 | 14.1176 |
| Maastricht univ | 7 | 22 | 85 | 34 | 708 | 20.8235 |
| Ohio state univ | 3 | 26 | 54 | 33 | 1414 | 42.8485 |
| Fudan univ | 4 | 18 | 27 | 33 | 525 | 15.9091 |
| Ucl | 2 | 24 | 34 | 32 | 1245 | 38.9062 |
| Univ amsterdam | 7 | 23 | 91 | 30 | 1452 | 48.4 |
| Stanford univ | 1 | 37 | 65 | 30 | 1455 | 48.5 |
| Univ louisville | 1 | 27 | 40 | 30 | 2079 | 69.3 |
| Peking univ | 4 | 10 | 18 | 30 | 666 | 22.2 |
| Univ pittsburgh | 4 | 16 | 21 | 29 | 2318 | 79.931 |
| Karolinska inst | 2 | 22 | 51 | 28 | 2440 | 87.1429 |
| Mayo clin | 1 | 30 | 41 | 28 | 842 | 30.0714 |
| Johns hopkins univ | 3 | 27 | 41 | 28 | 1617 | 57.75 |
| Inst gustave roussy | 5 | 15 | 25 | 28 | 1444 | 51.5714 |
| Washington univ | 6 | 27 | 59 | 27 | 996 | 36.8889 |
| Radboud univ nijmegen | 7 | 26 | 61 | 26 | 1159 | 44.5769 |
| Vrije univ amsterdam | 7 | 14 | 39 | 26 | 728 | 28 |
| Leiden univ | 7 | 18 | 66 | 25 | 1131 | 45.24 |
| Duke univ | 6 | 20 | 43 | 24 | 1013 | 42.2083 |
| Natl canc ctr | 3 | 13 | 29 | 24 | 1720 | 71.6667 |
| Univ oxford | 4 | 16 | 24 | 24 | 861 | 35.875 |
| Erasmus mc canc inst | 7 | 25 | 66 | 23 | 241 | 10.4783 |
| Aintree univ hosp nhs fdn trust | 2 | 27 | 48 | 23 | 4472 | 194.4348 |
| Inserm | 5 | 11 | 28 | 23 | 818 | 35.5652 |
| Univ oslo | 6 | 11 | 28 | 23 | 501 | 21.7826 |
| Univ milan | 5 | 5 | 13 | 21 | 629 | 29.9524 |
| Oslo univ hosp | 6 | 16 | 33 | 20 | 348 | 17.4 |
| Zhejiang univ | 4 | 12 | 18 | 20 | 262 | 13.1 |
| Sungkyunkwan univ | 3 | 6 | 15 | 20 | 419 | 20.95 |
| Med coll wisconsin | 1 | 28 | 54 | 19 | 837 | 44.0526 |
| Massachusetts gen hosp | 1 | 26 | 47 | 19 | 1041 | 54.7895 |
| Erasmus mc | 7 | 23 | 44 | 19 | 460 | 24.2105 |
| Emory univ | 1 | 25 | 40 | 19 | 429 | 22.5789 |
| Uppsala univ | 2 | 15 | 36 | 19 | 3006 | 158.2105 |
| Ludwig maximilians univ munchen | 2 | 15 | 24 | 19 | 239 | 12.5789 |
| Humanitas univ | 5 | 4 | 4 | 19 | 496 | 26.1053 |
| Charite univ med berlin | 2 | 11 | 33 | 18 | 166 | 9.2222 |
| Johns hopkins univ hosp | 1 | 18 | 26 | 18 | 686 | 38.1111 |
| Univ tokyo | 3 | 13 | 18 | 18 | 2003 | 111.2778 |
| Karolinska univ hosp | 2 | 18 | 34 | 17 | 360 | 21.1765 |
| Harvard med sch | 1 | 22 | 28 | 17 | 203 | 11.9412 |
| Univ hosp | 2 | 10 | 10 | 17 | 603 | 35.4706 |
| Yonsei univ | 3 | 4 | 6 | 17 | 439 | 25.8235 |
| Univ groningen | 7 | 13 | 37 | 16 | 564 | 35.25 |
| Cleveland clin | 6 | 17 | 21 | 16 | 496 | 31 |
| Hop paul brousse | 5 | 9 | 19 | 16 | 999 | 62.4375 |
| Harvard univ | 1 | 9 | 13 | 16 | 1085 | 67.8125 |
| Univ ulsan | 3 | 5 | 11 | 16 | 445 | 27.8125 |
| Univ saarland | 2 | 4 | 4 | 16 | 211 | 13.1875 |
| Univ maryland | 1 | 9 | 16 | 15 | 288 | 19.2 |
| Charite | 2 | 9 | 11 | 15 | 1232 | 82.1333 |
| Univ new s wales | 3 | 4 | 7 | 15 | 387 | 25.8 |
| Vanderbilt univ | 1 | 22 | 38 | 14 | 452 | 32.2857 |
| Univ calif los angeles | 1 | 20 | 28 | 14 | 815 | 58.2143 |
| Seoul natl univ | 3 | 17 | 23 | 14 | 196 | 14 |
| Royal marsden hosp | 2 | 20 | 21 | 14 | 555 | 39.6429 |
| Heidelberg univ | 2 | 7 | 17 | 14 | 488 | 34.8571 |
| European inst oncol | 5 | 8 | 16 | 14 | 410 | 29.2857 |
| Aarhus univ hosp | 6 | 8 | 11 | 14 | 447 | 31.9286 |
| Soochow univ | 4 | 1 | 1 | 14 | 387 | 27.6429 |
| Humboldt univ | 2 | 13 | 33 | 13 | 1711 | 131.6154 |
| Univ utrecht | 7 | 11 | 32 | 13 | 271 | 20.8462 |
| Med univ vienna | 2 | 16 | 22 | 13 | 2817 | 216.6923 |
| Univ penn | 1 | 14 | 16 | 13 | 589 | 45.3077 |
| Univ chicago | 6 | 11 | 16 | 13 | 1408 | 108.3077 |
| Univ munich | 2 | 8 | 13 | 13 | 695 | 53.4615 |
| City hope natl med ctr | 1 | 10 | 11 | 13 | 218 | 16.7692 |
| Nara med univ | 3 | 5 | 8 | 13 | 147 | 11.3077 |
| Huazhong univ sci & technol | 4 | 5 | 6 | 13 | 603 | 46.3846 |
| Univ florida | 6 | 16 | 26 | 12 | 681 | 56.75 |
| Univ paris 05 | 5 | 13 | 22 | 12 | 405 | 33.75 |
| German canc res ctr | 2 | 11 | 22 | 12 | 2475 | 206.25 |
| Univ paris saclay | 5 | 15 | 21 | 12 | 280 | 23.3333 |
| Univ rochester | 6 | 14 | 18 | 12 | 584 | 48.6667 |
| Aichi canc ctr hosp | 3 | 5 | 16 | 12 | 1432 | 119.3333 |
| Chinese acad med sci | 4 | 8 | 13 | 12 | 142 | 11.8333 |
| Univ bern | 2 | 7 | 13 | 12 | 181 | 15.0833 |
| St james univ hosp | 2 | 7 | 8 | 12 | 863 | 71.9167 |
| Kumamoto univ | 3 | 7 | 7 | 12 | 267 | 22.25 |
| Peking univ canc hosp & inst | 4 | 1 | 2 | 12 | 132 | 11 |
| Univ calif san diego | 1 | 27 | 37 | 11 | 303 | 27.5455 |
| Clin univ navarra | 5 | 23 | 29 | 11 | 188 | 17.0909 |
| Free univ berlin | 2 | 5 | 25 | 11 | 63 | 5.7273 |
| Hadassah hebrew univ | 1 | 10 | 14 | 11 | 733 | 66.6364 |
| Hosp clin barcelona | 5 | 8 | 14 | 11 | 303 | 27.5455 |
| Catholic univ korea | 3 | 5 | 14 | 11 | 84 | 7.6364 |
| Univ wisconsin | 1 | 9 | 11 | 11 | 328 | 29.8182 |
| Lund univ | 2 | 7 | 11 | 11 | 223 | 20.2727 |
| Univ turin | 3 | 8 | 10 | 11 | 233 | 21.1818 |
| H lee moffitt canc ctr & res inst | 1 | 7 | 9 | 11 | 394 | 35.8182 |
| Univ magdeburg | 5 | 7 | 9 | 11 | 502 | 45.6364 |
| Otto von guericke univ | 2 | 4 | 8 | 11 | 103 | 9.3636 |
| Yokohama city univ | 3 | 7 | 7 | 11 | 1226 | 111.4545 |
| Ctr leon berard | 5 | 3 | 7 | 11 | 257 | 23.3636 |
| Univ london imperial coll sci technol & med | 1 | 3 | 7 | 11 | 395 | 35.9091 |
| Shandong univ | 4 | 5 | 5 | 11 | 142 | 12.9091 |
| Goethe univ frankfurt | 2 | 2 | 2 | 11 | 1124 | 102.1818 |
| Sichuan univ | 4 | 1 | 1 | 11 | 91 | 8.2727 |
| Univ med ctr groningen | 7 | 19 | 61 | 10 | 206 | 20.6 |
| Amphia hosp | 7 | 15 | 46 | 10 | 115 | 11.5 |
| Sarah cannon res inst | 1 | 17 | 30 | 10 | 145 | 14.5 |
| Oregon hlth & sci univ | 6 | 14 | 29 | 10 | 278 | 27.8 |
| Yale sch med | 1 | 22 | 28 | 10 | 162 | 16.2 |
| Univ kentucky | 6 | 11 | 24 | 10 | 130 | 13 |
| Case western reserve univ | 6 | 15 | 18 | 10 | 289 | 28.9 |
| Hop europeen georges pompidou | 5 | 8 | 18 | 10 | 143 | 14.3 |
| Univ colorado | 1 | 16 | 17 | 10 | 519 | 51.9 |
| Tech univ munich | 2 | 9 | 17 | 10 | 1211 | 121.1 |
| Univ british columbia | 1 | 13 | 14 | 10 | 63 | 6.3 |
| Univ sydney | 3 | 11 | 14 | 10 | 565 | 56.5 |
| Univ miami | 1 | 9 | 12 | 10 | 446 | 44.6 |
| Yale univ | 1 | 7 | 12 | 10 | 113 | 11.3 |
| Univ pisa | 1 | 10 | 11 | 10 | 368 | 36.8 |
| Univ minnesota | 1 | 6 | 10 | 10 | 256 | 25.6 |
| St george hosp | 3 | 2 | 4 | 10 | 193 | 19.3 |
| Chinese peoples liberat army gen hosp | 4 | 1 | 1 | 10 | 335 | 33.5 |
| Univ padua | 1 | 1 | 1 | 10 | 227 | 22.7 |
| Med spectrum twente | 7 | 14 | 58 | 9 | 189 | 21 |
| Maxima med ctr | 7 | 15 | 55 | 9 | 118 | 13.1111 |
| Univ washington | 1 | 21 | 31 | 9 | 382 | 42.4444 |
| Carolinas med ctr | 1 | 17 | 25 | 9 | 447 | 49.6667 |
| Univ hosp gasthuisberg | 2 | 13 | 23 | 9 | 4095 | 455 |
| Univ med ctr | 2 | 18 | 20 | 9 | 1029 | 114.3333 |
| Univ hosp zurich | 6 | 12 | 20 | 9 | 163 | 18.1111 |
| Univ liverpool | 4 | 12 | 20 | 9 | 317 | 35.2222 |
| Univ med ctr hamburg eppendorf | 2 | 11 | 14 | 9 | 418 | 46.4444 |
| Capital med univ | 4 | 7 | 14 | 9 | 205 | 22.7778 |
| Gustave roussy | 5 | 7 | 12 | 9 | 296 | 32.8889 |
| Collaborat innovat ctr canc med | 4 | 4 | 12 | 9 | 75 | 8.3333 |
| Univ heidelberg hosp | 2 | 6 | 11 | 9 | 3263 | 362.5556 |
| Univ texas sw med ctr dallas | 6 | 6 | 10 | 9 | 432 | 48 |
| Univ hosp frankfurt | 2 | 9 | 9 | 9 | 216 | 24 |
| Inst bergonie | 5 | 7 | 8 | 9 | 399 | 44.3333 |
| Kleijnen systemat reviews ltd | 7 | 1 | 8 | 9 | 78 | 8.6667 |
| Univ athens | 3 | 3 | 3 | 9 | 225 | 25 |
| Biocompatibles uk ltd | 2 | 2 | 3 | 9 | 170 | 18.8889 |
| Queen elizabeth hosp | 5 | 2 | 2 | 9 | 480 | 53.3333 |

**Table S5.** The top 10 journals with the most published articles in this field

| **Element** | **Number of articles** | **Total citations** | **Average citations** | **Total link strength** |
| --- | --- | --- | --- | --- |
| *ANNALS OF SURGICAL ONCOLOGY* | 100 | 3396 | 33.96 | 948 |
| *CARDIOVASCULAR AND INTERVENTIONAL RADIOLOGY* | 74 | 2141 | 28.93 | 910 |
| *JOURNAL OF SURGICAL ONCOLOGY* | 63 | 1746 | 27.71 | 606 |
| *EJSO* | 60 | 1267 | 21.12 | 454 |
| *CANCERS* | 57 | 498 | 8.74 | 705 |
| *JOURNAL OF VASCULAR AND INTERVENTIONAL RADIOLOGY* | 52 | 2447 | 47.06 | 371 |
| *WORLD JOURNAL OF GASTROENTEROLOGY* | 48 | 1630 | 33.96 | 714 |
| *ANTICANCER RESEARCH* | 47 | 823 | 17.51 | 371 |
| *EUROPEAN RADIOLOGY* | 43 | 1549 | 36.02 | 383 |
| *HPB* | 43 | 1042 | 24.23 | 448 |

**Table S6.** Top 20 most influential authors according to the h-index.

| **Rank** | **Authors** | **h-index** | **g-index** | **m-index** | **Total citations** | **Number of articles** |
| --- | --- | --- | --- | --- | --- | --- |
| 1 | KEMENY NE | 30 | 56 | 1.875 | 3246 | 67 |
| 2 | D'ANGELICA MI | 29 | 49 | 1.8125 | 2470 | 61 |
| 3 | JARNAGIN WR | 29 | 52 | 1.8125 | 2725 | 61 |
| 4 | KINGHAM TP | 19 | 37 | 1.35714286 | 1383 | 44 |
| 5 | VERHOEF C | 16 | 28 | 1 | 867 | 41 |
| 6 | DEMATTEO RP | 28 | 37 | 1.75 | 2420 | 37 |
| 7 | ALLEN PJ | 22 | 37 | 1.46666667 | 1635 | 37 |
| 8 | SOFOCLEOUS CT | 21 | 37 | 1.3125 | 1668 | 37 |
| 9 | RICKE J | 20 | 37 | 1.25 | 3574 | 37 |
| 10 | SALEM R | 21 | 33 | 1.3125 | 1822 | 33 |
| 11 | PAWLIK TM | 19 | 31 | 1.1875 | 1459 | 31 |
| 12 | DE BAERE T | 16 | 29 | 1 | 1402 | 29 |
| 13 | MEIJERINK MR | 15 | 29 | 1 | 1148 | 29 |
| 14 | CERCEK A | 17 | 28 | 1.41666667 | 1231 | 28 |
| 15 | SOLOMON SB | 16 | 27 | 1 | 1573 | 27 |
| 16 | GONEN M | 16 | 26 | 1 | 1400 | 26 |
| 17 | VAUTHEY JN | 15 | 26 | 1.07142857 | 744 | 26 |
| 18 | LAM MGEH | 14 | 26 | 0.93333333 | 814 | 26 |
| 19 | ADAM R | 19 | 25 | 1.1875 | 4083 | 25 |
| 20 | MARTIN RCG | 19 | 24 | 1.188 | 1417 | 24 |

**Table S7.** The top 20 most frequently used keywords

| **Rank** | **keywords** | **Occurrences** | **Total link strength** |
| --- | --- | --- | --- |
| 1 | colorectal cancer | 911 | 1839 |
| 2 | liver metastases | 576 | 1197 |
| 3 | colorectal liver metastases | 357 | 585 |
| 4 | hepatectomy | 295 | 713 |
| 5 | metastases | 232 | 537 |
| 6 | radiofrequency ablation | 232 | 532 |
| 7 | hepatic | 161 | 383 |
| 8 | ablation | 158 | 401 |
| 9 | hepatic arterial infusion | 129 | 278 |
| 10 | radioembolization | 126 | 289 |
| 11 | chemotherapy | 125 | 323 |
| 12 | survival | 95 | 241 |
| 13 | microwave ablation | 92 | 239 |
| 14 | radiotherapy | 86 | 203 |
| 15 | sbrt | 86 | 170 |
| 16 | prognosis | 74 | 167 |
| 17 | sirt | 70 | 155 |
| 18 | yttrium-90 | 65 | 181 |
| 19 | oligometastases | 65 | 141 |
| 20 | irinotecan | 57 | 160 |

# Supplementary Figures


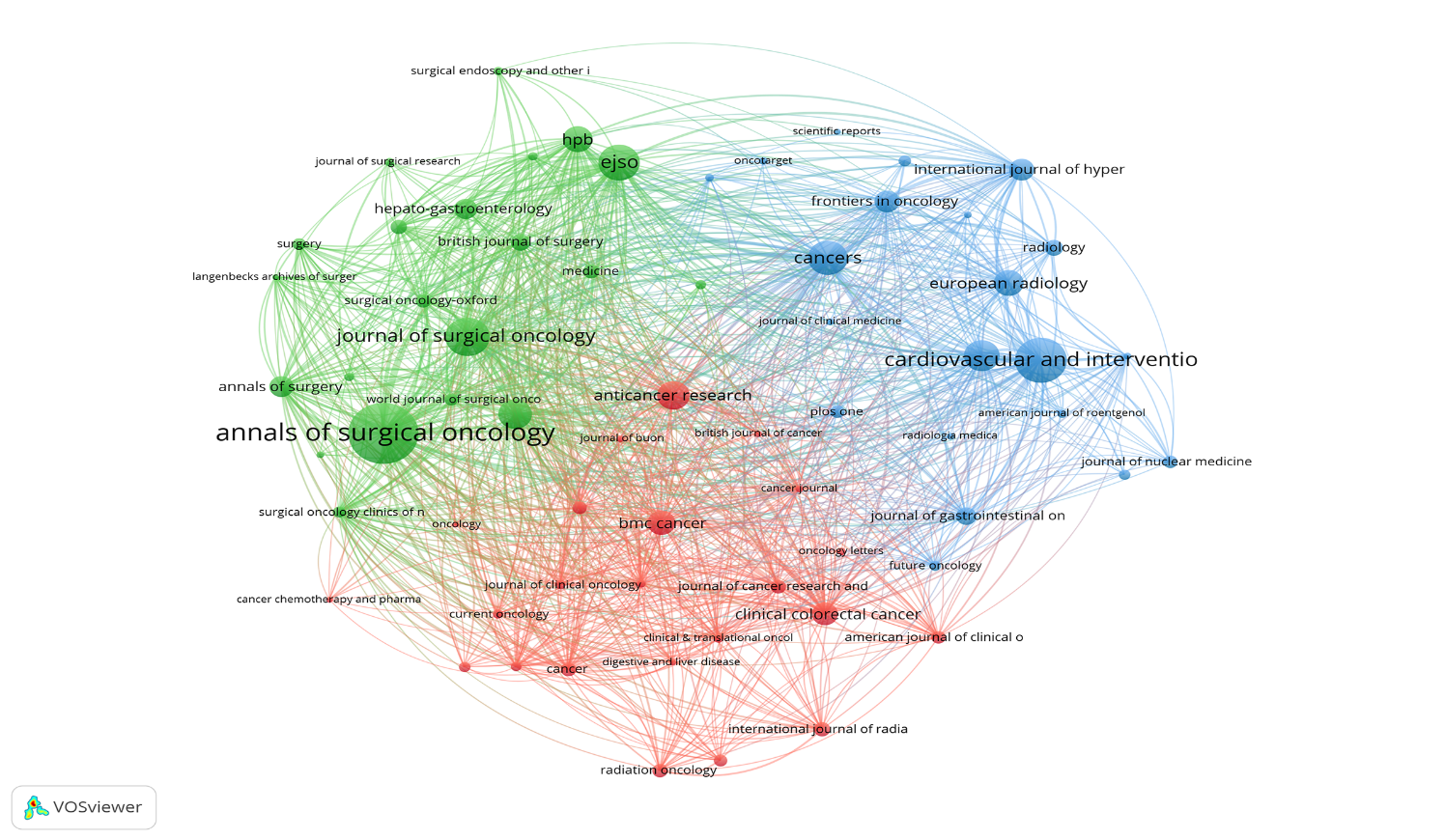


**Supplementary Figure 1.A network map of bibliographic coupling between sources.** The node size indicates the number of publications; the node color indicates the cluster to which the journal belongs; node correlation is determined by the number of references they share.


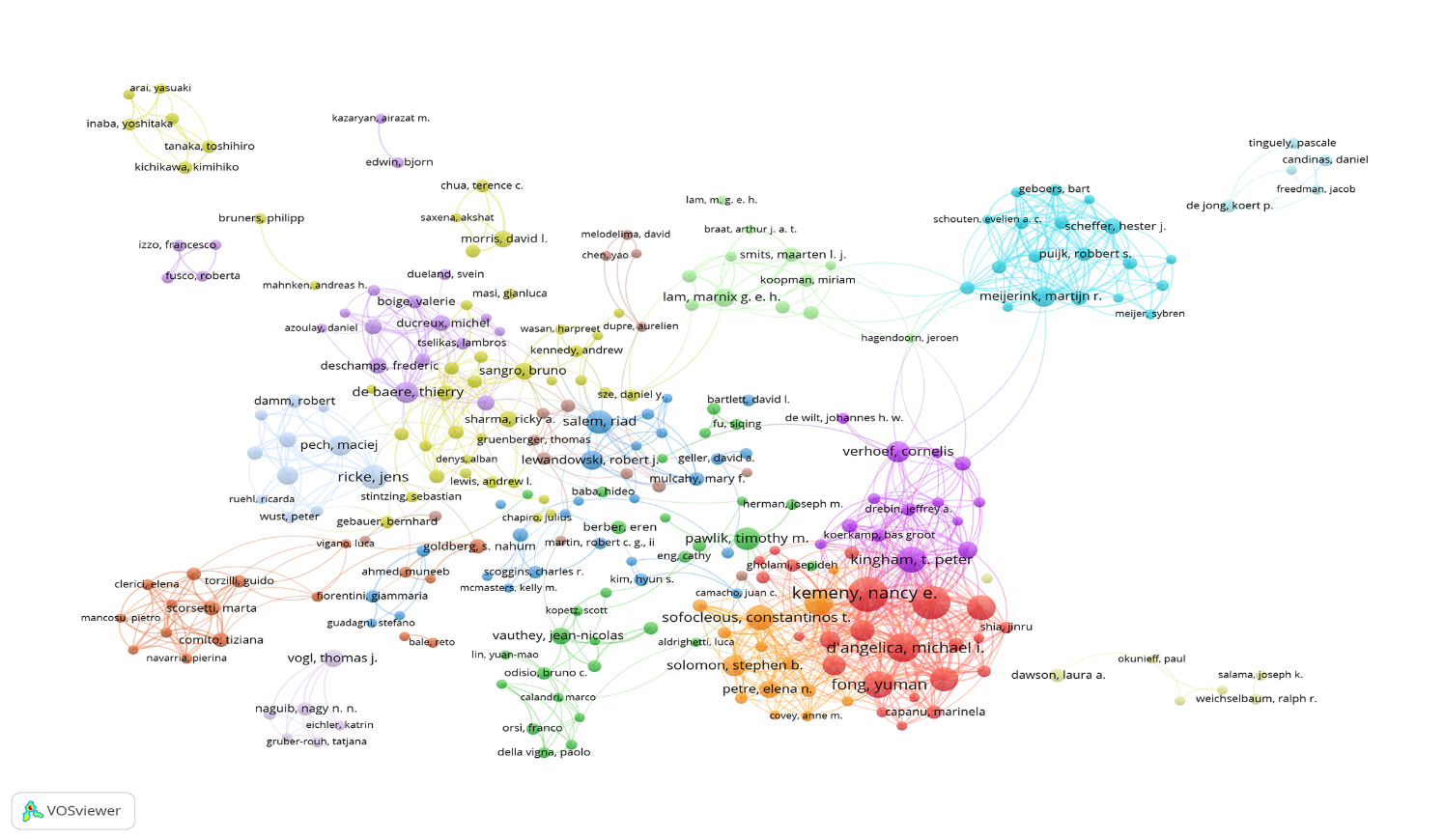


**Supplementary Figure 2. A network map of the co-authorship among authors.** The node size indicates the number of papers; the node color indicates the cluster to which the author belongs; the line between nodes indicates that there is a cooperative relationship.


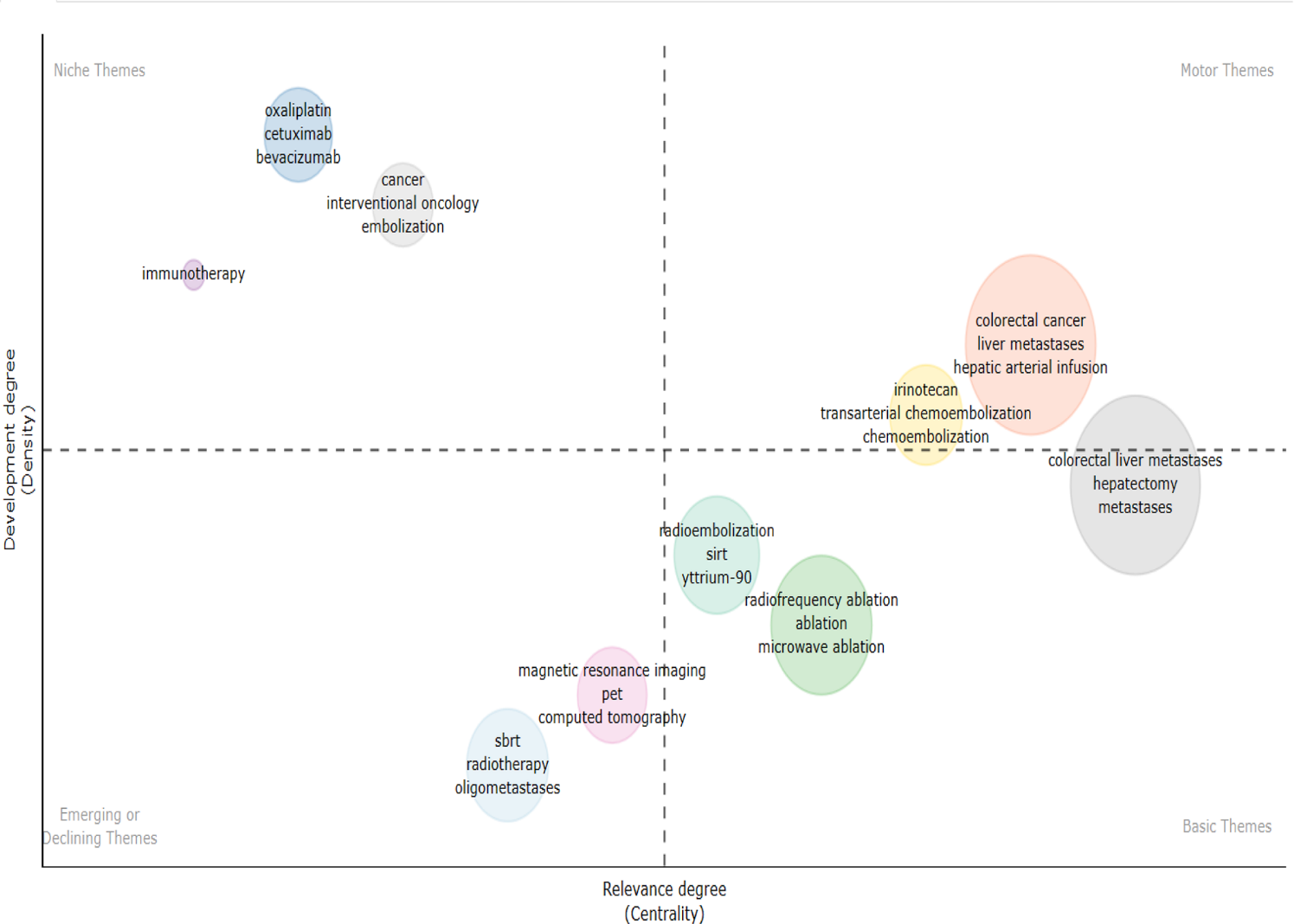


**Supplementary Figure 3. Analysis of thematic map.** The x-axis represents the centrality of keywords, while the y-axis represents density. Centrality indicates the degree of connection between clusters, while density represents the level of interconnectedness among keywords within a cluster.
